# The New 4-*O*-Methylhonokiol Analog GS12021 Inhibits Inflammation and Macrophage Chemotaxis: Role of AMP-Activated Protein Kinase α Activation

**DOI:** 10.1371/journal.pone.0117120

**Published:** 2015-02-23

**Authors:** Sora Kim, Sun-O Ka, Youngyi Lee, Byung-Hyun Park, Xiang Fei, Jae-Kyung Jung, Seung-Yong Seo, Eun Ju Bae

**Affiliations:** 1 College of Pharmacy, Woosuk University, Wanju-gun, Jeollabuk-do, Korea; 2 Department of Biochemistry, Medical School and Diabetes Research Center, Chonbuk National University, Jeonju, Jeollabuk-do, Korea; 3 College of Pharmacy, Gachon University, Incheon, Korea; 4 College of Pharmacy, Chungbuk National University, Cheongju, Korea; University of Oklahoma Health Science Center, UNITED STATES

## Abstract

Preventing pathologic tissue inflammation is key to treating obesity-induced insulin resistance and type 2 diabetes. Previously, we synthesized a series of methylhonokiol analogs and reported that compounds with a carbamate structure had inhibitory function against cyclooxygenase-2 in a cell-free enzyme assay. However, whether these compounds could inhibit the expression of inflammatory genes in macrophages has not been investigated. Here, we found that a new 4-*O*-methylhonokiol analog, 3′,5-diallyl-4′-methoxy-[1,1′-biphenyl]-2-yl morpholine-4-carboxylate (GS12021) inhibited LPS- or TNFα-stimulated inflammation in macrophages and adipocytes, respectively. LPS-induced phosphorylation of nuclear factor-kappa B (NF-κB)/p65 was significantly decreased, whereas NF-κB luciferase activities were slightly inhibited, by GS12021 treatment in RAW 264.7 cells. Either mitogen-activated protein kinase phosphorylation or AP-1 luciferase activity was not altered by GS12021. GS12021 increased the phosphorylation of AMP-activated protein kinase (AMPK) α and the expression of sirtuin (SIRT) 1. Inhibition of mRNA expression of inflammatory genes by GS12021 was abolished in AMPKα1-knockdown cells, but not in SIRT1 knockout cells, demonstrating that GS12021 exerts anti-inflammatory effects through AMPKα activation. The transwell migration assay results showed that GS12021 treatment of macrophages prevented the cell migration promoted by incubation with conditioned medium obtained from adipocytes. GS12021 suppression of p65 phosphorylation and macrophage chemotaxis were preserved in AMPKα1-knockdown cells, indicating AMPK is not required for these functions of GS12021. Identification of this novel methylhonokiol analog could enable studies of the structure-activity relationship of this class of compounds and further evaluation of its *in vivo* potential for the treatment of insulin-resistant states and other chronic inflammatory diseases.

## INTRODUCTION

Metabolic syndrome (including hyperlipidemia, fatty liver, and obesity-associated insulin resistance and type-2 diabetes mellitus (T2DM)) is an important problem worldwide. It is well established that adipose tissue is a mediator of inflammation and innate immunity [1, 2]. Therefore, strategies to curb inflammation of adipose tissue as therapy to treat metabolic syndrome have become popular.

In response to increased intake of energy, adipose tissue increases in mass due to hypertrophy (increase in cell size) and hyperplasia (increase in cell numbers). Expanded adipose tissue produces and secretes adipokines/chemokines such as tumor necrosis factor (TNF) α, interleukin (IL)-6, IL-1β, and monocyte chemoattractant protein-1 (MCP)-1. Pro-inflammatory cytokines secreted from adipose tissue act in an endocrine manner on peripheral tissues (e.g., skeletal muscle) and the liver, as well as on adipose tissue itself in an autocrine manner to disturb normal insulin signaling, thereby inducing insulin resistance. In adipose tissue, infiltrating macrophages and other immune cells as well as adipocytes are responsible for tissue inflammation. Infiltration of immune cells to adipose tissue is mediated primarily by chemokines secreted by adipose tissue itself. In particular, MCP-1 acts as a major chemokine to further recruit monocytes/macrophages into adipose tissue, leading to aggravation of inflammation in adipose tissue and systemic insulin resistance [3].

The phenolic neolignans honokiol and 4-*O*-methylhonokiol are the biologically active components of *Magnolia officinalis*. They have been shown to have anticancer, antifibrosis, antithrombotic, and anti-inflammatory effects in various cell types and animal models [4–10]. Recent studies have shown that supplementation with honokiol or 4-*O*-methylhonokiol ameliorates accumulation of body fat, insulin resistance, and adipose inflammation in high-fat-fed mice [11, 12]. The findings that honokiol and 4-*O*-methylhonokiol exert anti-inflammatory effects *in vitro* and that they prevent obesity, inflammation in adipose tissue, and insulin resistance in mice support the notion that macrophage-mediated inflammation of adipose tissue is a key mediator of insulin resistance and T2DM.

Previously, we designed and synthesized a series of derivatives of 4-*O*-methylhonokiol (including derivatives of aryl carbamate) to increase its biological activity and metabolic stability and reported their inhibitory activities against cyclooxygenase (COX)-2 enzyme in a cell-free system [13]. Among the 4-*O*-methylhonokiol analogs we tested, carbamate compounds showed more potent inhibitory activity against COX-2 than the selective COX-2 inhibitor celecoxib as well as the natural product honokiol. We also ascertained if carbamate compounds could prevent nitric oxide (NO) production in lipopolysaccharide (LPS)-stimulated macrophages, but few compounds were found to inhibit NO production at non-cytotoxic concentrations. However, whether 4-*O*-methylhonokiol analogs can inhibit the expression of pro-inflammatory genes and cytokine production in macrophages remains unknown.

Thus, in the present study, we hypothesized that 4-*O*-methylhonokiol analogs could inhibit the LPS-stimulated inflammatory response, and we examined the influences of these analogs on MCP-1 production and macrophage chemotaxis as well. We also investigated the molecular mechanism of action of a new 4-*O*-methylhonokiol analog, GS12021, which was chosen as a representative compound with anti-inflammatory and chemotaxis-inhibitory activities.

## MATERIALS AND METHODS

### Synthesis of 3′,5-diallyl-4′-methoxy-[1,1′-biphenyl]-2-yl morpholine-4-carboxylate (GS12021)

Triphosgene (89 mg, 0.3 mmol) maintained at 0°C was added to a CH_2_Cl_2_ solution (1 mL) of 4-*O*-methylhonokiol (42 mg, 0.15 mmol) and pyridine (80 mg, 1 mmol). After stirring for 2 h at ambient temperature, morpholine (26 mg, 0.3 mmol) was added to the reaction mixture. After stirring for 12 h at ambient temperature, the reaction mixture was diluted with CH_2_Cl_2_, washed with aqueous NH_4_Cl solution and brine, dried over MgSO_4_, and concentrated under reduced pressure. The residue was purified by flash column chromatography on silica gel (ethyl acetate:hexanes = 1:2) to afford the morpholinylcarbamate (GS12021) (40 mg, 68%). ^1^H-NMR (400 MHz, CDCl_3_) δ 7.23 (dd, 1H, *J* = 8.4 and 1.8 Hz), 7.18 (d, 1H, *J* = 1.2 Hz), 7.16 (d, 1H, *J* = 1.8 Hz), 7.15 (d, 1H, *J* = 8.4 and 1.8 Hz), 7.10 (d, 1H, *J* = 7.8 Hz), 6.88 (d, 1H, *J* = 8.4 Hz), 6.02–5.95 (m, 2H), 5.12–5.03 (m, 4H), 3.86 (s, 3H), 3.61 and 3.54 (two bs, 4H), 3.44 (bs, 4H), 3.41–3.39 (m, 4H); IR (neat) 2963, 2916, 2857, 1720, 1241, 1198 cm^-1^; ^13^C-NMR (150 MHz, CDCl_3_) δ 156.7, 153.6, 146.4, 137.6, 137.1, 136.7, 134.5, 130.8, 130.4, 130.1, 128.1, 128.0, 127.9, 123.0, 116.0, 115.5, 110.0, 66.6, 55.5, 44.7, 39.6, 34.4; LRMS (ESI) *m*/z 394 (M+H^+^) and 416 (M+Na^+^). The synthetic procedures for the remaining compounds (**c1~c9**) can be found in S1 File and Figure A in S1 File.

### Cell cultures

A murine macrophage cell line RAW 264.7 was maintained in growth medium containing Dulbecco’s modified Eagle’s medium (DMEM), 10% fetal bovine serum (FBS), 50 U/mL penicillin and 50 μg/mL streptomycin at 37°C in a humidified atmosphere with 5% CO_2_. Peritoneal macrophages were isolated from wild type or myeloid-specific SIRT1 knockout mice by peritoneal lavage 3 days after injection of 3 mL of 3% thioglycolate (Difco, Sparks, MD, USA) and plated in 12-well plates at 2 × 10^5^ cells/well. All experimental procedures were approved by the Institutional Animal Care and Use Committee of Chonbuk National University. Murine 3T3-L1 preadipocyte cells were maintained in growth medium containing DMEM, 10% FBS, 50 U/mL penicillin and 50 μg/mL streptomycin at 37°C in a humidified atmosphere with 10% CO_2_ and induced to differentiate as described previously [14].

### Cell viability

Cell viability was determined using the 3-(4,5-dimethylthiazol-2-yl)-2,5-diphenylthetrazolium bromide (MTT) assay. Cells in 96-well culture plates were incubated with compounds at 20 μM or 40 μM in the presence of FBS for 24 h. After incubation, 20 μL of MTT (0.5 mg/mL in PBS) was added to each well and the cells were incubated further for 3 h at 37°C. Formation of the violet precipitate formazan was monitored at 560 nm and 670 nm with a spectrophotometer.

### Measurement of NO levels

Production of NO was estimated by measuring the amount of nitrite (a stable metabolite of NO) using the Griess reagent, as described previously. Briefly, cells were pretreated with GS12021 or honokiol for 1 h before the addition of LPS. After 24 h, aliquots of culture supernatants were mixed with an equal volume of a modified Griess reagent comprising a 1:1 mixture of 1% sulfanilamide in 30% acetic acid and 0.1% N-(1-naphthyl)ethylenediamine dihydrochloride in 60% acetic acid, at room temperature for 5 min, and absorbance was measured at 540 nm using a spectrophotometer.

### Cell lysis and western blot analyses

Whole-cell lysate preparation and western blot analyses were performed as described previously [14]. Briefly, cells were lysed in buffer containing 10 mM Tris-HCl (pH 7.1), 100 mM NaCl, 1 mM EDTA, 10% glycerol, 0.5% Triton X-100, 0.5% Nonidet P-40, 1 mM dithiothreitol and 0.5 mM phenylmethylsulfonyl fluoride, supplemented with proteinase and phosphatase inhibitors. Protein concentrations in cell lysates were determined using a Bio-Rad Protein Assay (Bio-Rad Laboratories, Hercules, CA, USA). Aliquots of lysates underwent electrophoresis in 6–10% sodium dodecyl sulfate-polyacrylamide gels (20 μg of protein/lane). Separated proteins were transferred onto nitrocellulose membranes (GE Healthcare, Piscataway, NJ, USA). Membranes were blocked with 0.4% skimmed milk in TBS-1% Tween 20 and incubated with primary antibodies, followed by incubation with secondary antibodies. Immunoreactive proteins were visualized using an ECL Chemiluminescence Detection kit (Amersham Biosciences, Buckinghamshire, UK). Images were obtained using a ChemiDoc XRS+ system (Bio-Rad). The primary antibodies used were as follows: inducible nitric oxide synthase (iNOS) from BD Biosciences (Palo Alto, CA, USA); β-tubulin (#PA1–16947) from Thermo Scientific (Waltham, MA, USA); COX-2 (sc-1745) and inhibitor of kappa B alpha (IκBα; sc-371), from Santa Cruz Biotechnology (Santa Cruz, CA, USA); p-p65 (Ser536, ab76302) from Abcam (Cambridge, MA, USA); and phosphorylated c-Jun N-terminal kinase (p-JNK; #92515), p-p38 (#4631), phosphorylated extracellular signal-regulated kinase (p-ERK; #9101), p-Akt (#92759), phosphorylated AMP protein kinase (p-AMPK; #2535), AMPKα (#27153), p-acetyl CoA carboxylase (p-ACC; #36615), and ACC (#3662) from Cell Signaling Technology (Beverly, MA, USA).

### RNA isolation and real-time reverse-transcription polymerase chain reaction (RT-qPCR)

Total RNA was extracted from cells or adipose tissue with TRIzol reagent (Invitrogen Carlsbad, CA, USA). Total RNA (2 μg) was reverse-transcribed using random primers (Promega, Madison, WI, USA). RT-qPCR was performed as described previously using an ABI7000 and Stratagene3000 MXP PCR cycler with Sybr Green Detection System 6. mRNA expression of all the genes tested was normalized to Rps3 expression. All the RT-qPCR experiments were replicated three times with duplicate analysis. Primer sequences (sense and antisense, respectively) were as follows: *Nos2*, 5′-AATCTTGGAGCGAGTTGTGG-3′ and 5′-CAGGAAGTAGGTGAGGGCTTG-3′ *Il6*, 5′-CCAGAGATACAAAGAAATGATGG-3′ and 5′-ACTCCAGAAGACCAGAGGAAAT-3′; *Mcp1/Ccl2*, 5′-TCTGGACCCATTCCTTCTTG-3′ and 5′-AGGTCCCTGTCATGCTTCTG-3′ *Tnfa*, 5′-GCCACCACGCTCTTCTGCCT-3′ and 5′-GGCTGATGGTGTGGGTGAGG-3′, *COX2*, 5′-AAAGCATCTGGCCTACACCC-3′ and 5′-ATGCTACCTTTGCACGGTCA-3′, *Il1b*, 5′-AAATACCTGTGGCCTTGGGC-3′ and 5′-CTTGGGATCCACACTCTCCAG-3′.

### RNA interference

Duplexes of small interfering RNA (siRNA), targeting AMPKα1 mRNA (target sequences: 5′-CGGGAUCCAUCAGCAACUA-3′ for sense and 5′-UAGUUGCUGAUGGAUCCCG-3′ for antisense) and a negative control (scrambled sequence) were purchased from Dharmacon Research (Lafayette, CO, USA). We transfected 2 × 10^6^ RAW 264.7 cells with 0.5 nmol of siRNA oligonucleotides to mouse AMPKα1 or scrambled control siRNA using Lipofectamine 2000 (Invitrogen). siRNA-transfected cells were incubated for 24 h at 37°C before assays.

### Transient transfection and luciferase reporter assay

RAW 264.7 cells were seeded on 24-well plates and incubated for 24 h. For reporter gene assays, cells were transfected with 200 ng of nuclear factor-kappa B (NF-κB)-luciferase (luc) or AP-1 luciferase (Stratagene, La Jolla, CA, USA), and 20 ng of pRL-TK (Promega) using Lipofectamine 2000 (Invitrogen). After 24 h of incubation, cells were treated with vehicle or GS12021 1 h before LPS (10 ng/mL) exposure and incubated for a further 24 h. Protein was extracted from cells using passive lysis buffer (Promega), and luciferase activity was measured using a Dual Luciferase Reporter assay (Promega) from Lumat LB 9507 (Berthold, Bad Wildbad, Germany).

### Macrophage chemotaxis assay

For the preparation of adipocyte-conditioned medium (CM), mature 3T3-L1 adipocytes (> 90% of cells showing large lipid droplets when observed under a microscope) were used. At day 10 of differentiation, culture media were changed and the cells were incubated further with migration media (serum-free, 0.2% endotoxin-free, and free fatty acid-free bovine serum albumin in DMEM) for 48 h. The media collected were centrifuged at 15,000 × *g* for 10 min to remove cell debris, and the supernatants collected were aliquoted and frozen at -70°C until use for the chemotaxis assay. For migration *per se*, 5 × 10^5^ RAW 264.7 cells suspended in migration media were placed in the upper chamber of a polycarbonate filter with a pore size of 8 μm (24-transwell format; Corning, Lowell, MA, USA), whereas adipocyte-CM was placed in the lower chamber. After 3 h of migration, cells were fixed in formalin and stained with 0.1% crystal violet. Macrophages that had remained in the upper chamber were removed by wiping the filters with cotton tips. Macrophages found on the bottom of filters were counted as cells that had carried out chemotaxis. Cells were quantified from 5 fields/condition; each condition was carried out in triplicate.

### Statistical analyses

Data are provided as mean ± SEM values. The significance of differences between treatment groups was determined by one-way ANOVA with the Turkey’s post-hoc test using GraphPad Prism v4.0 (GraphPad, San Diego, CA, USA). *P* < 0.05 or 0.01 was considered significant.

## RESULTS

### 4-*O*-Methylhonokiol analogs suppressed LPS-stimulated expression of iNOS and COX-2

**Fig 1:**
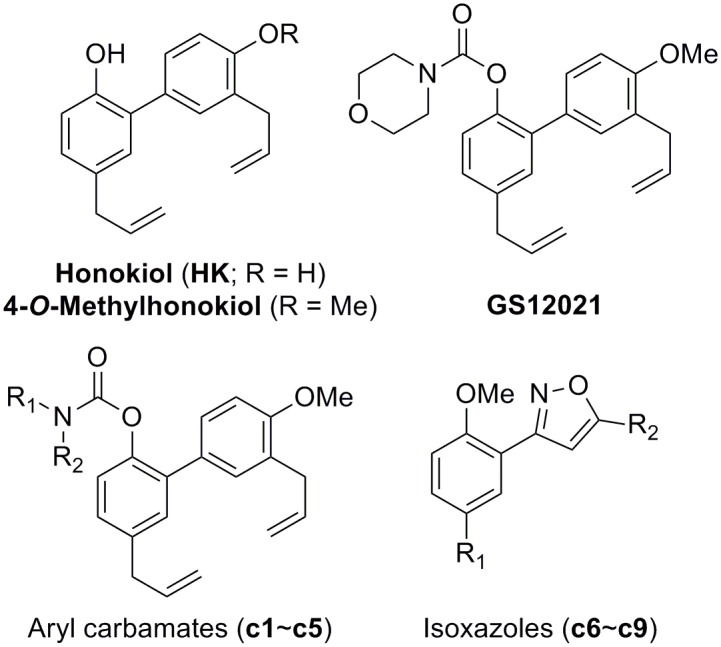
Chemical structures of honokiol, 4-*O*-methylhonokiol, and their derivatives.

**Fig 2:**
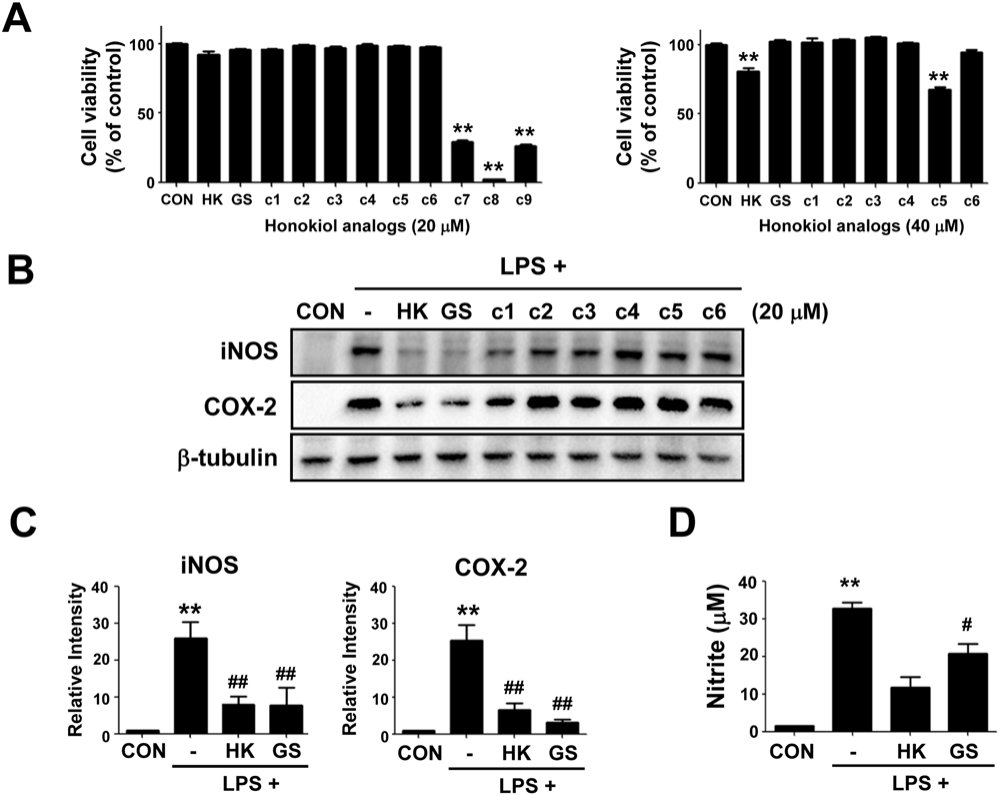
Effects of treatment with a honokiol analog on cell viability, expression of iNOS and COX-2, and production of NO in RAW 264.7 cells. (A) RAW 264.7 cells were incubated with each compound (20 μM or 40 μM) in the presence of serum for 24 h. Cell viability was determined by the MTT assay. (B)-(C) Cells were pretreated with 20 μM of compounds 1 h before LPS treatment (10 ng/mL) for 24 h. The protein level was measured by western blotting. The experiments were repeated at least three times and representative blots (B) and quantification data (C) are shown. β-tubulin was used as a loading control. (D) Nitrite content. Nitrite levels were measured by spectrophotometric means with Griess reagent using supernatant media obtained from the cells in (B). HK, honokiol; GS, GS12021; c1–c9, arbitrary names for the synthetic honokiol analogs. Data are provided as mean ± SEM values. *P < 0.05, **P < 0.01 *versus* CON; ^#^ *P* < 0.05, ^##^ *P* < 0.01 *versus* LPS alone treatment.

We synthesized a series of honokiol derivatives (including methylhonokiol analogs) according to the methods described in the Methods and Materials section and S1 File. The chemical structures of the compounds are shown in Fig. 1 and Figure A in S1 File. Previously, honokiol was reported to have pro-apoptotic or anti-proliferative effects in cancer cells. Hence, we first determined the effects of compounds on the viability of RAW 264.7 cells. Cells were incubated with the agents (20 μM or 40 μM) for 24 h in the presence of serum and cell viability determined by the MTT assay. Treatment of cells with each of the compounds c7, c8, or c9 at 20 μM reduced cell viability markedly (Fig. 2A). When cells were treated with 40 μM of each compound, cell viability was not affected except for honokiol and c5 (which reduced cell viability significantly). Therefore, subsequent experiments were conducted at 20 μM of each compound unless stated otherwise.

Next, we examined the effects of agents on LPS-induced expression of iNOS and COX-2 because honokiol has been reported to inhibit expression of pro-inflammatory genes. RAW 264.7 cells were pretreated with 20 μM of honokiol or its analogs 1 h before treatment with LPS (10 ng/mL) for 24 h. The protein level was measured by western blotting (representative blots were shown in Fig. 2B). As expected, honokiol treatment of RAW 264.7 cells resulted in inhibition of the expression of iNOS and COX-2. More importantly, one of the methylhonokiol analogs, GS12021, which has a novel structure, inhibited expression of iNOS and COX-2 with a potency equal to that seen with honokiol. Compounds between c1 and c7 did not affect expression of these genes. The effects of honokiol and GS12021 on the expression of iNOS and COX-2 as well as nitrite production are shown in Fig. 2C and D. Taken together, our observations indicated that GS12021 was as efficacious as honokiol in preventing gene expression of iNOS and COX-2 and that GS12021 was not cytotoxic, whereas honokiol showed cytotoxicity at 40 μM. Therefore, we chose GS12021 as a representative methylhobokiol analog for further study.

### GS12021 inhibits the expression of pro-inflammatory genes and cytokines/chemokine secretion in RAW 264.7 cells

**Fig 3:**
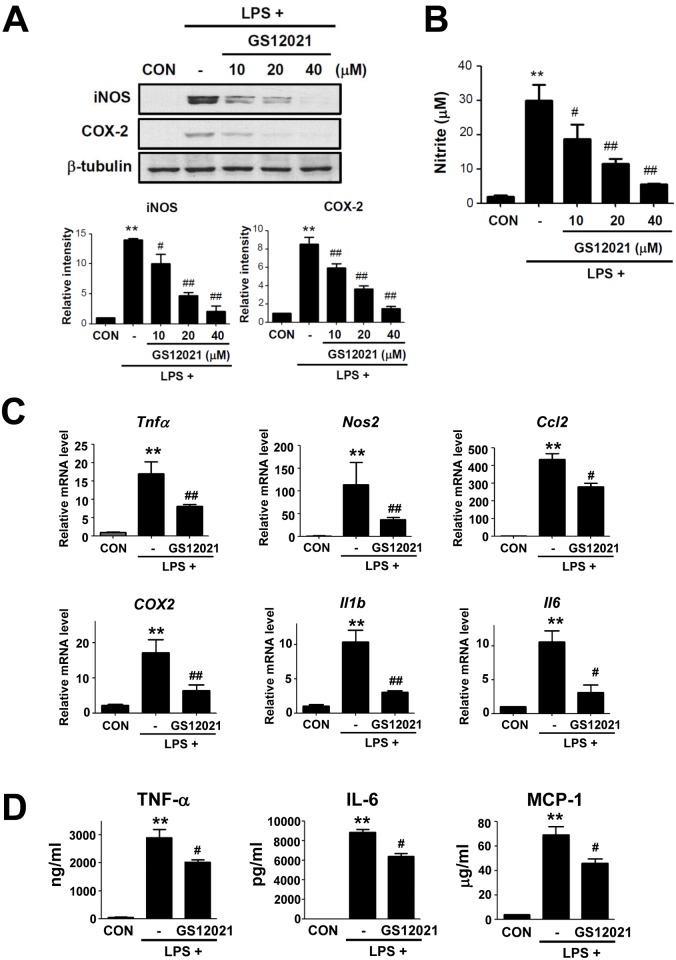
Anti-inflammatory effect of GS12021 in RAW 264.7 cells. (A) Concentration-dependent effect of GS12021 on expression of iNOS and COX-2. Representative western blot images and quantification data are shown. Cells were pretreated with the indicated concentrations of GS12021 1 h before LPS treatment (10 ng/mL) for 24 h. (B) Nitrite production in cells from (A). (C) mRNA expression of inflammatory genes/cytokines. Cells were treated with agents for 6 h and RT-qPCR was performed. (D) Cytokine levels in supernatant media from cells treated with LPS ± GS12021 for 6 h (TNFα) or 24 h (IL-6 and MCP-1). All the experiments were repeated at least three times and the data are provided as mean ± SEM values. ** *P* < 0.01 *versus* CON; ^#^ *P* < 0.05, ^##^ *P* <0.01 *versus* LPS treatment alone.

We observed that GS12021 significantly suppressed expression of iNOS and COX-2 proteins; therefore, we analyzed whether the inhibition was dependent upon concentration. GS12021 significantly inhibited the LPS-stimulated induction of iNOS and COX-2 at 10, 20, and 40 μM in a concentration-dependent manner (Fig. 3A). Consistently, LPS-stimulated NO production was suppressed by pretreatment with GS12021 (Fig. 3B). To confirm the anti-inflammatory effect of GS12021 in macrophages, we measured the mRNA level of inflammatory genes/chemokines in GS12021-treated cells by RT-qPCR. mRNA levels of TNFα, iNOS, MCP-1, COX-2, IL-1β and IL-6 were strongly induced by LPS treatment, whereas pretreatment with GS12021 markedly blocked their expression (Fig. 3C). Secretion of TNFα and IL-6, as demonstrated by the enzyme-linked immunosorbent assay (ELISA), was also suppressed by GS12021 pretreatment (Fig. 3D). Secretion of MCP-1 (a representative chemokine essential for macrophage chemotaxis) was also reduced significantly by GS12021 pretreatment, implying that GS12021 may exhibit anti-chemotactic effects as well as anti-inflammatory effects in macrophages.

### GS12021 inhibits macrophage chemotaxis

**Fig 4:**
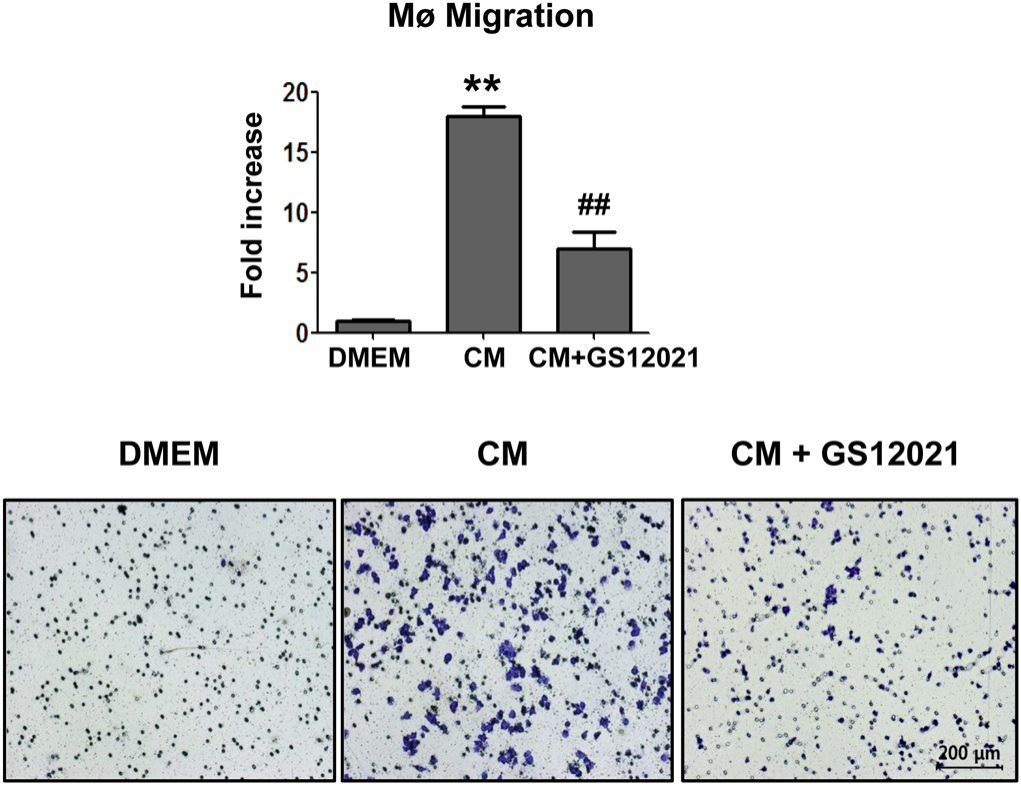
Macrophage chemotaxis was markedly inhibited by GS12021 treatment. Macrophage migration assays using DMEM (migration media) or conditioned medium (CM) collected from adipocytes were conducted. RAW 264.7 cells were incubated with DMSO or GS12021 at 20 μM for 3 h and detached cells used for the migration assay in the presence of DMEM or CM in the lower well. Cells placed in transwells were incubated for 3 h for migration, and migrated cells found on the lower parts of transwells were counted after staining with crystal violet. The experiments were repeated three times and the representative microscope images for migrated cells are shown below at ×100 magnification. **P < 0.01 *versus* DMEM; ^##^ *P* < 0.01 *versus* CM.

We performed an in vitro chemotaxis assay to investigate the functional consequences of the inhibitory activity of GS12021 on macrophage inflammation [14,15]. RAW 264.7 macrophages were treated with GS12021 for 3 h and then seeded on the upper insert well of a chemotaxis chamber and incubated for 3 h in the presence of DMEM or adipocyte-CM in the lower chamber. A 3-h migration was chosen because it provides optimal results in the macrophage chemotaxis assay, as described previously [15]. Adipocyte-CM significantly stimulated macrophage chemotaxis in comparison with DMEM (Fig. 4). However, exposure of macrophages to GS12021 abrogated cell migration in response to adipocyte-CM, indicating that GS12021 inhibited the ability of macrophages to migrate toward adipocyte-derived chemoattractants.

### GS12021 inhibits an NF-κB/p65 phosphorylation in RAW 264.7 cells

**Fig 5:**
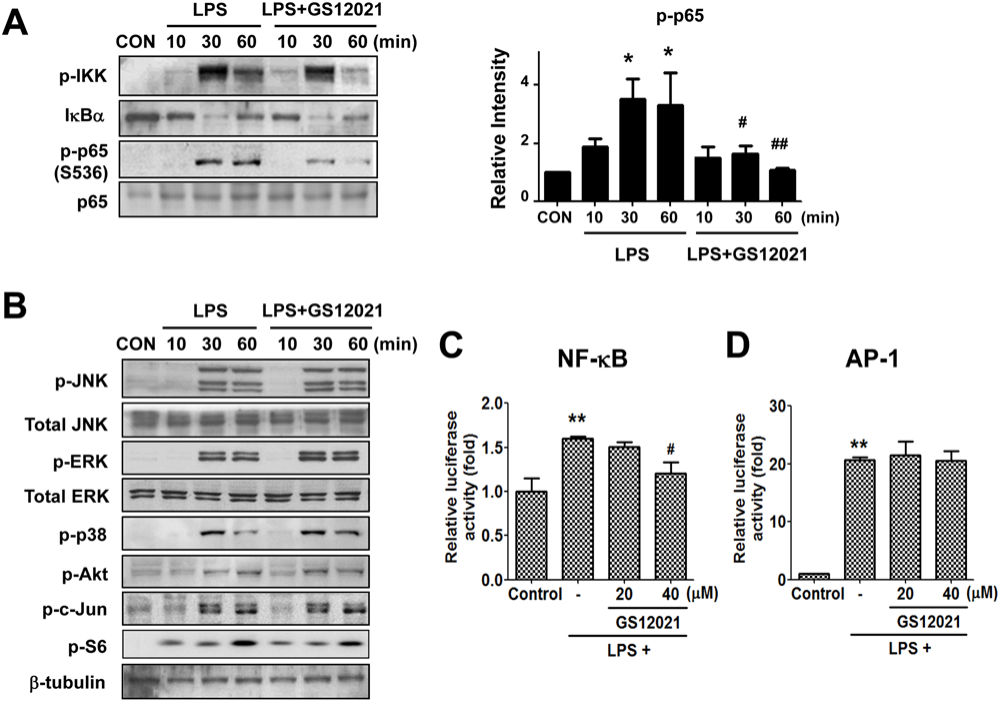
Effect of GS12021 treatment on LPS-stimulated cell signaling in RAW 264.7 cells. Immunoblot analyses of phosphorylated IKK, IκBα and phosphorylated- and total-p65. The experiments were repeated at least three times, and the representative images and the densitometry results for phosphorylated p65 are shown in (A). Phosphorylations of JNK, ERK, p38 MAPK, Akt, c-Jun and S6 protein (B). Cells were pretreated with vehicle or GS12021 (20 μM for 1 h) and stimulated with LPS for the time periods indicated. (C) NF-κB reporter assay (N = 4). (D) AP-1 reporter assay (N = 4). RAW 264.7 cells were transfected with NF-κB or AP-1 luciferase reporter plasmid and, after 24 h, cells were incubated with GS12021 (20 μM or 40 μM) for a further 24 h. **P < 0.01 *versus* CON; ^##^ *P* < 0.05 *versus* LPS alone.

Studies have reported that honokiol inhibits inflammatory responses through blockade of NF-κB signaling [4,10]. To elucidate the anti-inflammatory mechanism of action of the newly identified methylhonokiol analog GS12021, we examined the effect of GS12021 on signaling pathways activated by LPS in RAW 264.7 cells. I kappa B kinase (IKK) phosphorylation and IκBα degradation were enhanced by LPS but were not changed by GS12021 pretreatment (Fig. 5A). Activation of the NF-κB component RelA/p65 requires post-translational modifications; therefore, we examined the phosphorylation of p65. Interestingly, although GS12021 pretreatment did not cause any alteration in IKK phosphorylation and IκBα degradation stimulated by LPS, GS12021 pretreatment resulted in significant blockade of phosphorylation of p65 (Fig. 5A). Moreover, the phosphorylations of JNK, ERK, p38 MAPK, Akt, c-Jun and S6 protein, all of which have been shown to regulate inflammatory responses, were not inhibited by GS12021 pretreatment (Fig. 5B).

Considering our finding that GS12021 attenuates NF-κB phosphorylation, we examined the influence of GS12021 on NF-κB promoter activity using the NF-κB luciferase assay. In NF-κB luciferase plasmid-transfected RAW 264.7 cells, luciferase activity was stimulated by LPS treatment for 24 h but was decreased by pretreatment with GS12021 at 40 μM (Fig. 5C). The promoter activity of AP-1, another important regulator of inflammation, was not changed by GS12021 treatment (Fig. 5D). Because the ability of GS12021 at 20 μM to inhibit NF-κB luciferase activity was mild even though the degree of inhibition of protein expression of iNOS was almost complete (Figs. 2B and 3A), we hypothesized that GS12021 may affect the stability of iNOS protein. Therefore, an additional study was conducted to ascertain if GS12021 treatment altered the stability of iNOS protein. RAW 264.7 cells were incubated with LPS for 6 h to induce iNOS expression with or without GS12021 (20 μM) pretreatment for 0.5 h and were then exposed to cycloheximide (5 μg/mL), an inhibitor of protein translation. Exposure of cells to cycloheximide for up to 4 h resulted in a gradual decrease in the iNOS protein level, which was not altered in the presence of GS12021 (Figure B in S1 File). Our observations indicate that GS12021 attenuated the expression of pro-inflammatory genes at the level of transcription and its regulation of transcription is at least in part associated with the inhibition of NF-κB/p65 phosphorylation.

### GS12021 activates an AMPKα signaling pathway in RAW 264.7 cells

**Fig 6:**
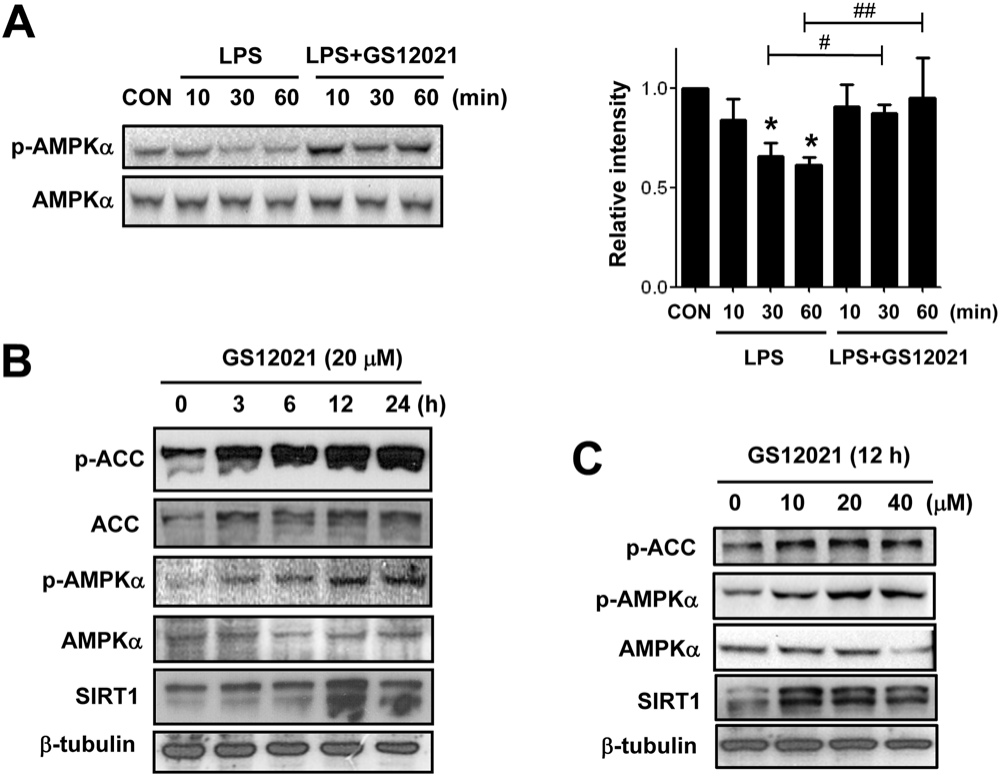
Effect of GS12021 treatment on AMPKα activation and SIRT1 expression in RAW 264.7 cells. **(A)** Levels of phosphorylated AMPKα and total AMPKα were measured by western blotting. Cells were pretreated with vehicle or GS12021 (20 μM for 1 h) and stimulated with LPS for the time periods indicated. Quantification results are shown in the right panel. *P < 0.05 *versus* CON; ^#^ *P* < 0.05, ^##^ *P* <0.01 *versus* LPS alone. (B) Cells were treated with GS12021 (20 μM) for the indicated time periods and western blotting was performed with specific antibodies against p-ACC, p-AMPKα, and SIRT1. β-tubulin was used as a loading control. (C) Concentration-dependent effect of GS12021 on AMPK activation and SIRT1 expression. Cells were treated with different concentrations of GS12021 for 12 h, and western blotting analyses were performed.

AMPKα is a cellular energy sensor protein and is known to modulate inflammation [16–18]. Honokiol is known to activate AMPKα in cancer cells to mediate its antitumor effect [19]; therefore, we examined the effects of GS12021 on AMPKα signaling to further explore its anti-inflammation mechanism in macrophages. LPS treatment in RAW 264.7 cells resulted in a decrease in AMPKα phosphorylation at 30 min and 60 min, which was in agreement with the finding that pro-inflammatory stimuli such as LPS can suppress AMPKα signaling [20]. However, GS12021 pretreatment increased AMPKα phosphorylation significantly (Fig. 6A). Consistently, time-course experiments showed that GS12021 significantly increased phosphorylation of ACC (a downstream substrate of AMPKα) from 3 h up to 24 h, as well as AMPKα phosphorylation (Fig. 6B). AMPK activity is known to be regulated by nicotinamide adenosine dinucleotide-dependent histone deacetylase sirtuin (SIRT)-1 as well as by upstream kinases such as liver kinase B 1 or calcium/calmodulin-dependent protein kinase kinase 1 [21]. Furthermore, SIRT1 has been reported to play a central part in the regulation of the pathogenesis of chronic inflammatory diseases [22]. Hence, we also examined SIRT1 expression after GS12021 treatment. Interestingly, SIRT1 expression was also increased by GS12021 treatment, implying that AMPKα activation and the stimulated SIRT1 expression might contribute to the anti-inflammatory effect of GS12021. The concentration-dependent effect of GS12021 on AMPKα activation and SIRT1 is shown in Fig. 6C.

### The anti-inflammatory effect of GS12021 is mediated by AMPKα but not by SIRT1

**Fig 7:**
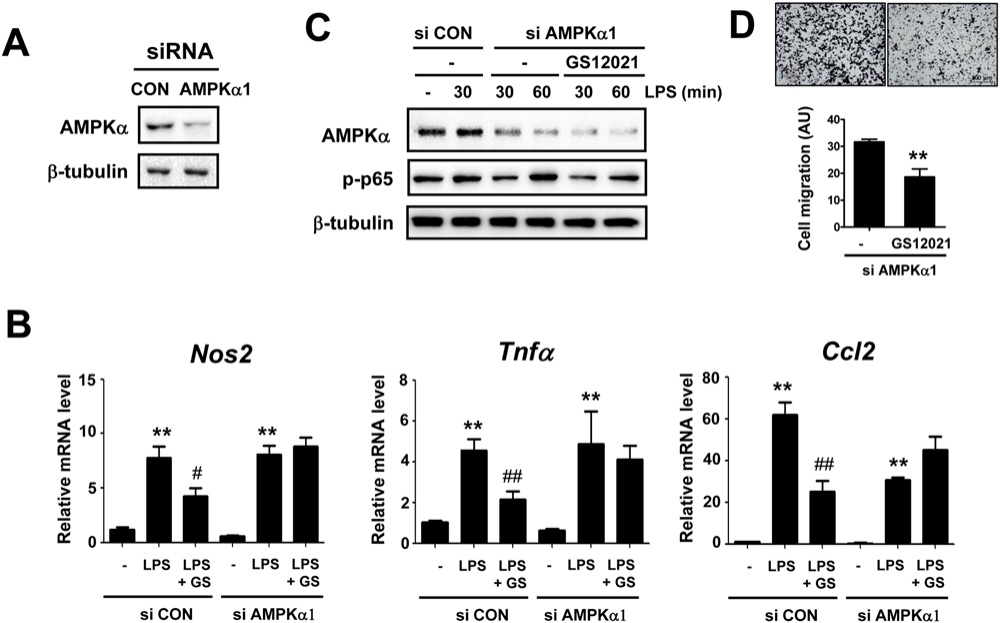
Anti-inflammatory effect, but not anti-chemotaxis effect, of GS12021 was attenuated in AMPKα1 knockdown cells. RAW 264.7 cells were transfected with siRNA of control (CON) or AMPKα1 and incubated for 24 h. Cells were subsequently treated with LPS or GS12021+LPS and mRNA or protein levels were determined. (A) AMPKα expression after siRNA transfection. (B) mRNA levels of iNOS, TNFα, and MCP-1/CCL2 in AMPKα1 knockdown macrophages. **P < 0.01 *versus* vehicle; ^#^ *P* < 0.05, ^##^ *P* < 0.01 *versus* LPS alone. (C)-(D) The effect of AMPKα1 knockdown on the GS12021 suppression of p-p65 and the adipocyte CM-mediated macrophage chemotaxis. Representative microphotographs of cell migration are shown above the quantification results (N = 3). AU means arbitrary units. **P < 0.01 *versus* vehicle.

To investigate the causal relationships between AMPKα activation or SIRT1 expression and macrophage inflammation by GS12021, we examined GS12021 responses in AMPKα knockdown or SIRT1 knockout cells. AMPKα1 is the predominant AMPKα isoform expressed by macrophages [20]; therefore, we performed an siRNA interference study using siRNA targeting AMPKα1. After transient transfection of siRNA targeting AMPKα1 or control siRNA in RAW 264.7 cells, cells were treated with LPS for 6 h with or without GS12021. The level of AMPKα was reduced by transfection of AMPKα1 siRNA (Fig. 7A). As expected, GS12021 treatment significantly inhibited the LPS-induced expression of inflammatory genes such as iNOS, TNFα, and MCP-1 in control siRNA transfected cells (Fig. 7B). However, in AMPKα knockdown cells, GS12021 had no effect on the inhibition of mRNA expression of inflammatory genes, indicating that AMPKα is necessary for GS12021 to inhibit LPS-induced inflammation. In an effort to confirm that the anti-inflammatory effect of GS12021 is mediated by AMPKα activation, we employed the AMPKα-specific chemical inhibitor compound C. Treatment with compound C alone could completely inhibit the expression of inflammatory genes by LPS treatment (data not shown). Given that many researchers have demonstrated that AMPKα activation plays an anti-inflammatory role, the anti-inflammatory effect of compound C in macrophages was unexpected and merits further investigation. Next, we examined the effect of siRNA for AMPKα1 on GS12021-inhibition of p65 phosphorylation. As shown in Fig. 7C, GS12021 was able to inhibit LPS-induced p65 phosphorylation in AMPKα1 depleted cells. Moreover, GS12021 also inhibited adipocyte CM stimulated cell migration in AMPKα1 depleted macrophages (Fig. 7D). These results indicate that AMPK was not required for GS12021 to inhibit either p65 phosphorylation or macrophage chemotaxis, being in a clear contrast to the anti-inflammatory function of GS12021.

**Fig 8:**
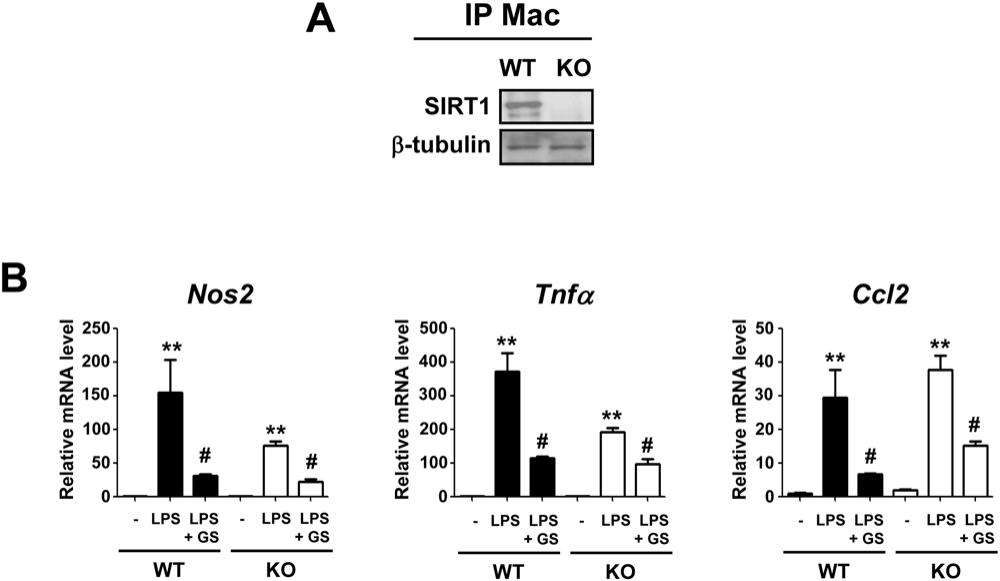
Anti-inflammatory effect of GS12021 was preserved in SIRT1 knockout (KO) macrophages. (A) SIRT1 expression in peritoneal macrophages obtained from wild-type (WT) or myeloid-specific SIRT1 KO mice. Peritoneal macrophages were isolated from WT or SIRT1 KO mice as described in the Materials and Methods section. (B) mRNA levels of inflammatory genes measured by RT-qPCR in macrophages from WT or SIRT1 KO mice. Cells were pretreated with GS12021 for 1 h, and then treated with LPS (10 ng/mL) for 6 h. ** *P* < 0.01 *versus* CON, # *P*<0.05 ## *P*<0.01 *versus* LPS alone.

Next, we tested the effect of GS12021 in SIRT1 knockout macrophages. To obtain peritoneal macrophages, we used the thioglycollate-elicited peritoneal macrophage protocol described in detail in the Materials and Methods section in wild-type (WT) and myeloid-specific SIRT1 knockout mice. SIRT1 appeared to be dispensable for the inhibition of inflammation by GS12021 (Fig. 8). In addition, methylhonokiol has been shown to activate peroxisome proliferator-activated receptor (PPAR) γ [23] and PPARγ plays an important role in the suppression of inflammation; therefore, we conducted an experiment using the PPARγ antagonist GW9662. Pretreating cells with GW9662 (10 μM) for 30 min before incubating the cells with GS12021 and LPS for 6 h did not reverse the GS12021-induced inhibition of iNOS, TNFα and MCP-1 mRNA expression (data not shown). Our observations indicate that activation of SIRT1 or PPARγ is not involved in the anti-inflammatory function of GS12021.

### GS12021 inhibits TNFα stimulated adipocyte inflammation

**Fig 9:**
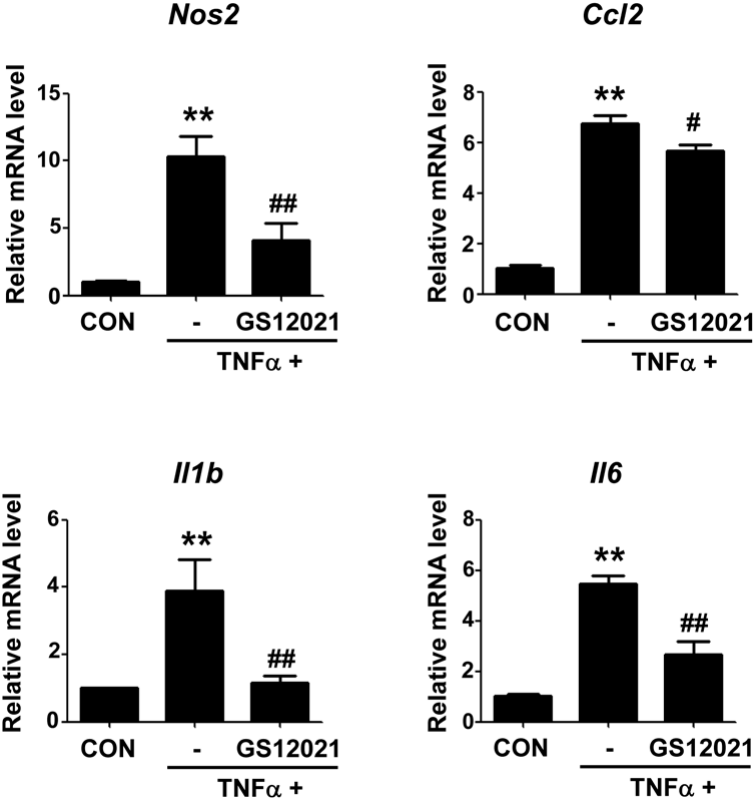
The effect of GS12021 treatment on the adipocyte inflammation. Fully differentiated 3T3-L1 adipocytes were treated with TNFα (10 ng/ml) for 6 h in the presence or absence of GS12021 (20 μM). mRNA levels of iNOS, MCP-1/CCL2, IL-1β and IL-6 were measured by RT-qPCR (N = 3). **P < 0.01 *versus* CON; ^#^ *P* < 0.05, ^##^ *P* < 0.01 *versus* TNFα alone.

Inhibition of inflammation is closely related to improvement of insulin resistance in diet-induced obesity. In view of the fact that adipose tissue inflammation in obesity results from the feed-forward inflammatory responses between adipocytes and macrophages, and to clearly determine the potential of GS12021 to curb obesity linked insulin resistance, we next examined whether GS12021 inhibits inflammation in adipocytes as in macrophages. Because the expression level of LPS receptor toll-like receptor 4 is very low in adipocytes, we treated cells with TNFα to induce inflammatory responses in adipocytes. As shown in Fig. 9, the mRNA expression of a series of inflammatory genes was promoted by TNFα treatment for 6 h in 3T3-L1 adipocytes but was blocked by GS12021 pretreatment, indicating an anti-inflammatory activity of GS12021 in adipocytes as well as in macrophages.

## DISCUSSION

Pathological tissue inflammation is a key process that can elicit and precipitate various conditions, including obesity and T2DM. Obesity develops gradually, and expansion of adipose tissue occurs due to the hypertrophy and hyperplasia of adipocytes. Subsequent increases in the release of pro-inflammatory adipokines/chemokines such as TNFα and MCP-1 as well as free fatty acids from adipocytes promote inflammation of adipose tissue and lead to insulin resistance in peripheral tissues. In particular, MCP-1, which is secreted from adipocytes and infiltrating macrophages in adipose tissue, acts as a major chemokine to further recruit monocytes/macrophages into adipose tissue, leading to aggravation of inflammation of adipose tissue.

Plasma levels of MCP-l increase in sepsis and obesity in humans [24–26]. MCP-1 (also known as CCL2) is a member of the C-C chemokine family and a potent chemotactic factor for monocytes [27]. MCP-1 is produced by various cell types, and monocyte/macrophages are the major source of this chemokine. MCP-1 mediates its effects through its receptor CCR2 and regulates the migration and infiltration of immune cells, thereby serving as a mediator of tissue inflammation. In addition to its chemotactic activity for leukocytes, MCP-1 plays a part in the metastasis and angiogenesis of tumors. It also plays a role in modulation of the proliferation and apoptosis of and protein synthesis in cells. Thus, MCP-1 is a potential “intervention point” for the treatment of various inflammatory diseases, such as multiple sclerosis, rheumatoid arthritis, atherosclerosis, and insulin-resistant T2DM [3], [28–32].

We identified a new 4-*O*-methylhonokiol analog, GS12021, which has potent anti-inflammatory functions in macrophages without affecting cell viability at ≤ 40 μM (the maximal concentration that we tested). Considering that honokiol reduced cell viability at 40 μM and that the anti-inflammatory efficacy of GS12021 was equivalent to that of honokiol at lower concentrations, GS12021 could be a superior option to honokiol in the treatment of various inflammatory diseases. More importantly, we showed that GS12021 opposed not only the production of the major chemokine MCP-1 and pro-inflammatory cytokines but also macrophage chemotaxis toward adipocyte-CM. It is well established that prevention of macrophage chemotaxis into peripheral tissues (e.g., adipose tissue) is a major therapeutic approach against chronic inflammatory conditions such as obesity and T2DM, highlighting the therapeutic importance of the findings of the present study.

In addition, we found that GS12021 inhibits LPS-stimulated NF-κB/p65 phosphorylation without affecting IκBα degradation. In unstimulated cells, the NF-κB p65/p50 heterodimer is held inactive in the cytoplasm by the inhibitory protein IκBα. Pro-inflammatory stimuli activate IKK, which in turn phosphorylates IκBα, resulting in its ubiquitination-mediated degradation, allowing the NF-κB released to enter the nucleus and activate gene expression. Thus, IκBα degradation is used widely as an indication of NF-κB activation. However, the transcriptional activity of NF-κB is also affected by post-translational modifications such as phosphorylation and acetylation, and these mechanisms are independent from IκBα degradation [33]. We found that GS12021 (20 μM) significantly inhibited the phosphorylation of p65 NF-κB at Ser536, an event required for the transactivation activity of NF-κB [34–36], but it did not prevent the degradation of IκBα stimulated by LPS. We also found that GS12021 significantly inhibited LPS-stimulated NF-κB luciferase activity only at 40 μM. Taken together, these findings suggest that GS12021 inhibits NF-κB and it has an anti-inflammatory function at high concentration, but at low concentration GS12021 may also have an anti-inflammatory role, independent of NF-κB inhibition. The present study showed that silencing of AMPKα1 expression by siRNA transfection impaired the ability of GS12021 to protect against LPS-induced mRNA expression of inflammatory genes. This finding established AMPK as a primary mediator of the anti-inflammatory actions of GS12021 on macrophages. SIRT1 was found to be dispensable for the anti-inflammatory actions of GS12021, which was demonstrated in the SIRT1-deficient macrophage study.

**Fig 10:**
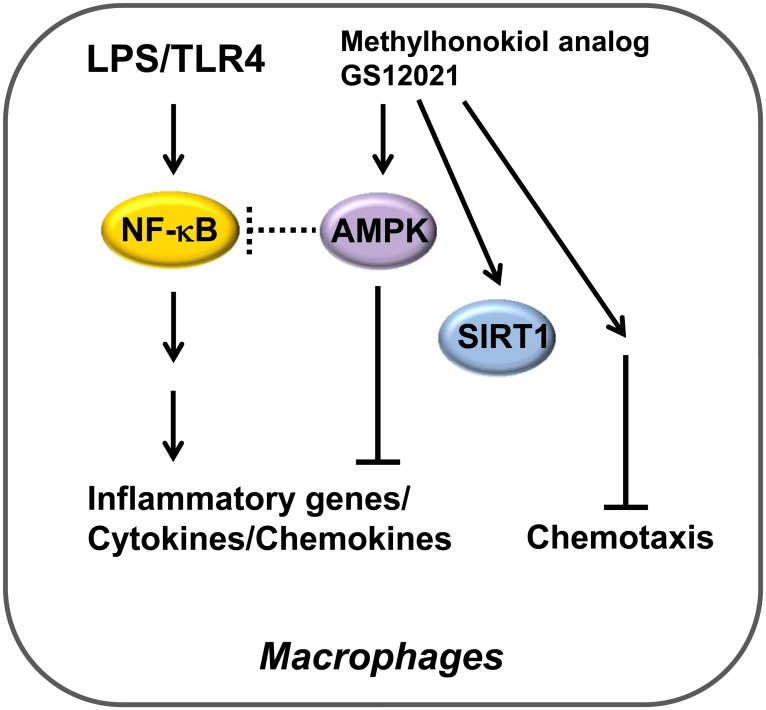
Graphical abstract shows that AMPK is required for GS12021 inhibition of inflammation by but not for its inhibition of chemotaxis in macrophages.

AMPK is a multimeric serine/threonine kinase comprising α-, β-, and γ-subunits and acts as a “sensor” of the energy status of cells. A wide range of environmental stressors that cause a reduction in the ATP/AMP ratio in cells serve to activate AMPK via multiple mechanisms, including phosphorylation of the α-subunit on Thr^172^ by upstream kinases. Activated AMPK then “shuts-off” anabolic pathways while simultaneously activating catabolic pathways. AMPK signaling is also critical for various physiologic processes, including inflammation as well as the proliferation and death of cells. Many studies have demonstrated that activation of AMPK signaling downregulates the function of the NF-κB system [37–39]. It seems that AMPK suppresses NF-κB signaling indirectly via its downstream mediators (e.g., SIRT1, Forkhead box O family, and PPARγ co-activator 1α, which can subsequently repress the expression of inflammatory factors). GS12021 increased the expression of SIRT1, and SIRT1 is a well-established modulator of inflammation through the deacetylation and transactivation of NF-κB, but our data demonstrated that SIRT1 is not necessary for the inhibitory function of GS12021 upon inflammation. Collectively, our data suggest that a new methylhonokiol analog GS12021 activates AMPK, which mediates GS12021’s anti-inflammatory function independently of SIRT1 (Fig. 10). We have tried many ways to address the question that how AMPK mediates GS12021’s anti-inflammatory function independently of NF-κB or SIRT1 with no conclusive results. Further additional studies are required to dissect the molecular mechanism of action of GS12021.

## CONCLUSION

We have identified a new 4-*O*-methylhonokiol analog, GS12021 and provided evidence that it exerts its anti-inflammatory effects in macrophages by activating AMPK signaling pathways. Given that there is an increasing need for safe and efficient treatment for T2DM and other chronic inflammatory diseases, our finding that GS12021 has potent anti-inflammatory effects in macrophages and adipocytes, and markedly inhibits macrophage chemotaxis in response to adipocyte-derived chemoattractants sheds light on therapies against obesity and obesity-associated insulin resistance. Further investigations of the effects of GS12021 *in vivo* will help to ascertain if GS12021 has therapeutic potential for obesity-linked inflammatory diseases and insulin resistance.

## Supporting Information

S1 File File contains Figures A and B. **Figure A**. Synthesis and structures of *O*-methylhonokiol derivatives. (A) Synthesis of aryl carbamate derivatives (GS12021 and **c1~c5**) from 4-*O*-methylhonokiol. (B) The structures of isoxazole derivatives (**c6**~**c9**) of 4-*O*-methylhonokiol. **Figure B**. The effect of GS12021 on iNOS protein stability. RAW 264.7 cells were incubated with LPS (10 ng/mL) for 6 h with or without pretreatment with GS12021 (20 μM) for 0.5 h, and then exposed to cycloheximide (CHX; 5 μg/mL) from 1 h to 4 h. Representative image of western blot analyses for iNOS expression.
(DOCX)
