# Supplementary material for: The New 4-O-Methylhonokiol Analog GS12021 Inhibits Inflammation and Macrophage Chemotaxis: Role of AMP-Activated Protein Kinase α Activation
Source: PLoS One. 2015 Feb 23;10(2):e0117120. doi: 10.1371/journal.pone.0117120 (PMC4338227; doi:10.1371/journal.pone.0117120)
Supplement: S1 File — Figure A. Synthesis and structures of O-methylhonokiol derivatives. (A) Synthesis of aryl carbamate derivatives (GS12021 and c1~c5) from 4-O-methylhonokiol. (B) The structures of isoxazole derivatives (c6~c9) of 4-O-methylhonokiol. Figure B. The effect of GS12021 on iNOS protein stability. RAW 264.7 cells were incubated with LPS (10 ng/mL) for 6 h with or without pretreatment with GS12021 (20 μM) for 0.5 h, and then exposed to cycloheximide (CHX; 5 μg/mL) from 1 h to 4 h. Representative image of western blot analyses for iNOS expression. (DOCX) [file pone.0117120.s001.docx]

**SuppORTING information**

**Materials and general methods**

All starting materials and reagents were obtained from commercial suppliers and were used without further purification. Air and moisture sensitive reactions were performed under an argon atmosphere. Flash column chromatography was performed using silica gel 60 (230-400 mesh, Merck) with the indicated solvents. Thin-layer chromatography was performed using 0.25 mm silica gel plates (Merck). ^1^H and ^13^C NMR spectra were recorded on either a Bruker 600 MHz or a JEOL 400 MHz spectrometer as solutions in deuteriochloroform (CDCl_3_) or methanol-d4. ^1^H NMR data were reported in the order of chemical shift, multiplicity (s, singlet; d, doublet; t, triplet; m, multiplet and/or multiple resonances), number of protons, and coupling constant (*J*) in hertz (Hz). Low resolution mass spectra were obtained on an Waters LCMS system (Waters 2489 UV/Visible Detector, Waters 3100 Mass, Waters 515 HPLC pump, SunFire C18 column 4.6 × 50 mm, 5 μm particle size, Waters 2545 Binary Gradient Module, Waters Reagent Manager, and Waters 2767 Sample Manager) with electrospray ionization.

**Synthetic procedures**

The synthesis of 4-*O*-methylhonokiol derivatives is shown in Figure A. Two aryl carbanate analogs (**c4** and **c5**) as well as four isoxazole analogs (**c6**~**c9**) were prepared by our previous report.^S^^[[1]](#footnote-1)^ Similarly, 4-*O*-methylhonokiol was used as starting material and treated with triphosgene and an appropriate amine such as morpholine, glycine methyl ester, and glycinamide to afford the resulting aryl carbamates (**GS12021**, **c1** and **c2**) in moderate yields. In the other hand, 4-fluorophenylcarbamate (**c3**) was given by the treatment of 4-*O*-methylhonokiol with 4-fluorophenyl isocyanate and pyridine.

**Methyl 2-((((3',5-diallyl-4'-methoxy-[1,1'-biphenyl]-2-yl)oxy)carbonyl)amino)acetate (c1).**

To a CH_2_Cl_2_ solution (1 mL) of 4-*O*-methylhonokiol (42 mg, 0.15 mmol) and pyridine (80 mg, 1 mmol) was added triphosgene (89 mg, 0.3 mmol) at 0 °C. After stirring for 2 h at ambient temperature, glycine methyl ester (14 mg, 3.0 mmol) was added to the reaction mixture. After stirring for 12 h at ambient temperature, the reaction mixture was diluted with CH_2_Cl_2_, washed with aqueous NH_4_Cl solution and brine, dried over MgSO_4_ and concentrated under reduced pressure. The residue was purified by flash column chromatography on silica gel (ethyl acetate : hexanes = 1 : 2) to afford the (2-methoxy-2-oxoethyl)carbamate **c1** (26 mg, 44%). ^1^H-NMR (400 MHz, CDCl_3_) δ 7.23 - 7.06 (m, 5H), 6.85 (d, 1H, *J* = 8.7 Hz), 5.99 - (m, 2H), 5.35 (bt, 1H, *J* = 5.2 Hz), 5.09 (m, 4H), 3.91 (d, 2H, *J* = 5.3 Hz), 3.8 (S, 3H), 3.70 (S, 3H), 3.36 (d, 4H, *J* = 6.3 Hz); IR (neat) 2954, 2312, 1739, 1487, 1247, 1197 cm^-1^; LRMS (ESI) *m/z* 396 (M+H^+^) and 418 (M+Na^+^).

**3',5-diallyl-4'-methoxy-[1,1'-biphenyl]-2-yl (2-amino-2-oxoethyl)carbamate (c2).**

To a CH_2_Cl_2_ solution (1 mL) of 4-*O*-methylhonokiol (42 mg, 0.15 mmol) and pyridine (80 mg, 1 mmol) was added triphosgene (89 mg, 0.3 mmol) at 0 °C. After stirring for 2 h at ambient temperature, glycinamide (22 mg, 0.3 mmol) was added to the reaction mixture. After stirring for 12 h at ambient temperature, the reaction mixture was diluted with CH_2_Cl_2_, washed with aqueous NH_4_Cl solution and brine, dried over MgSO_4_ and concentrated under reduced pressure. The residue was purified by flash column chromatography on silica gel (ethyl acetate : hexanes = 1 : 2) to afford the (2-amino-2-oxoethyl)carbamate **c2** (32 mg, 57%). ^1^H-NMR (400 MHz, CDCl_3_) δ 7.22 (m, 5H), 6.91 (d, 1H, *J* = 8.3 Hz), 6.03 (m, 2H), 5.19 (t, 1H, *J* = 5.5 Hz), 5.14 - 5.03 (m, 4H), 4.08 (d, 2H, *J* = 5.8 Hz), 3.85 (s, 3H), 3.42 (d, 4H, *J* = 5.8 Hz); IR (neat) 3742, 2972, 1514 cm^-1^; LRMS (ESI) *m/z* 381 (M+H^+^) and 403 (M+Na^+^).

**3',5-diallyl-4'-methoxy-[1,1'-biphenyl]-2-yl (4-fluorophenyl)carbamate (c3).**

To a CH_2_Cl_2_ solution (1 mL) of 4-*O*-methylhonokiol (42 mg, 0.15 mmol) and pyridine (80 mg, 1 mmol) was added 4-fluorophenyl chloroformate (52 mg, 0.3 mmol) at 0 °C. After stirring for 2 h at ambient temperature, the reaction mixture was diluted with CH_2_Cl_2_, washed with aqueous NH_4_Cl solution and brine, dried over MgSO_4_ and concentrated under reduced pressure. The residue was purified by flash column chromatography on silica gel (ethyl acetate : hexanes = 1 : 5) to afford the 4-fluorophenylcarbamate **c3** (35 mg, 59%). ^1^H-NMR (400 MHz, CDCl_3_) δ 7.37 (m, 5H), 7.09 (m, 2H), 6.99 (d, 1H, *J* = 8.3 Hz) 6.95 (bs, 1H), 6.14 (m, 2H), 5.25 (m, 4H), 3.93 (s, 3H), 3.53 (d, 2H, *J* = 6.3 Hz), 3.49 (d, 2H, *J* = 6.8 Hz); IR (neat) 1723, 1512, 1192 cm^-1^; LRMS (ESI) *m/z* 418 (M+H^+^) and 440 (M+Na^+^).

**Figure A.** Synthesis and structures of *O*-methylhonokiol derivatives. (A) Synthesis of aryl carbamate derivatives (GS12021 and **c1~c5**) from 4-*O*-methylhonokiol. (B) The structures of isoxazole derivatives (**c6**~**c9**) of 4-*O*-methylhonokiol.

**A**

**B**

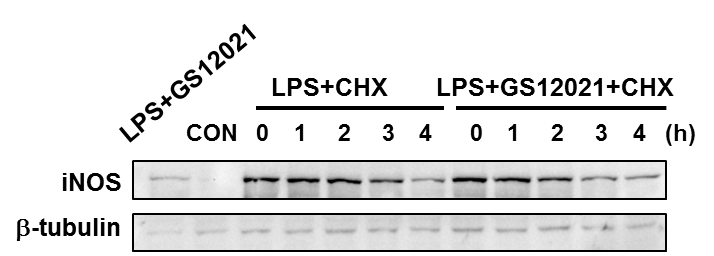
**Figure B.** The effect of GS12021 on iNOS protein stability. RAW 264.7 cells were incubated with LPS (10 ng/mL) for 6 h with or without pretreatment with GS12021 (20 μM) for 0.5 h, and then exposed to cycloheximide (CHX; 5 μg/mL) from 1 h to 4 h. Representative image of western blot analyses for iNOS expression.

1. (S1) Lee B, Kwak JH, Huang SW, Jang JY, Lim S, et al. (2012) Design and synthesis of 4-O-methylhonokiol analogs as inhibitors of cyclooxygenase-2 (COX-2) and PGF_1_ production. Bioorg Med Chem 20: 2860-2868. [↑](#footnote-ref-1)
